# Hematologic safety of ^177^Lu-PSMA-617 radioligand therapy in patients with metastatic castration-resistant prostate cancer

**DOI:** 10.1186/s13550-021-00805-7

**Published:** 2021-07-03

**Authors:** Daniel Groener, Cam Tu Nguyen, Justus Baumgarten, Benjamin Bockisch, Karen Davis, Christian Happel, Nicolai Mader, Christina Nguyen Ngoc, Jennifer Wichert, Severine Banek, Philipp Mandel, Felix K. H. Chun, Nikolaos Tselis, Frank Grünwald, Amir Sabet

**Affiliations:** 1 Department of Nuclear Medicine, University Hospital Frankfurt, Theodor Stern Kai 7, 60590 Frankfurt, Germany; 2 Department of Urology, University Hospital Frankfurt, Frankfurt, Germany; 3 Department of Radiation Oncology, University Hospital Frankfurt, Frankfurt, Germany

**Keywords:** PSMA, ^177^Lu-PSMA-617, Hematologic adverse events, Hematotoxicity, Metastatic castration-resistant prostate cancer

## Abstract

**Background:**

Myelosuppression is a potential dose-limiting factor in radioligand therapy (RLT). This study aims to investigate occurrence, severity and reversibility of hematotoxic adverse events in patients undergoing RLT with ^177^Lu-PSMA-617 for metastatic castration-resistant prostate cancer (mCRPC). The contribution of pretreatment risk factors and cumulative treatment activity is taken into account specifically.

**Methods:**

RLT was performed in 140 patients receiving a total of 497 cycles. A mean activity of 6.9 $\pm$ 1.3 GBq ^177^Lu-PSMA-617 per cycle was administered, and mean cumulative activity was 24.6 $\pm$ 15.9 GBq. Hematological parameters were measured at baseline, prior to each treatment course, 2 to 4 weeks thereafter and throughout follow-up. Toxicity was graded based on Common Terminology Criteria for Adverse Events v5.0.

**Results:**

Significant (grade ≥ 3) hematologic adverse events occurred in 13 (9.3%) patients, with anemia in 10 (7.1%), leukopenia in 5 (3.6%) and thrombocytopenia in 6 (4.3%). Hematotoxicity was reversible to grade ≤ 2 through a median follow-up of 8 (IQR 9) months in all but two patients who died from disease progression within less than 3 months after RLT. Myelosuppression was significantly more frequent in patients with pre-existing grade 2 cytopenia (OR: 3.50, 95%CI 1.08–11.32, *p* = 0.04) or high bone tumor burden (disseminated or diffuse based on PROMISE miTNM, OR: 5.08, 95%CI 1.08–23.86, *p* = 0.04). Previous taxane-based chemotherapy was associated with an increased incidence of significant hematotoxicity (OR: 4.62, 95%CI 1.23–17.28, *p* = 0.02), while treatment with ^223^Ra-dichloride, cumulative RLT treatment activity and activity per cycle were not significantly correlated (*p* = 0.93, 0.33, 0.29).

**Conclusion:**

Hematologic adverse events after RLT have an acceptable overall incidence and are frequently reversible. High bone tumor burden, previous taxane-based chemotherapy and pretreatment grade 2 cytopenia may be considered as risk factors for developing clinically relevant myelosuppression, whereas cumulative RLT activity and previous ^223^Ra-dichloride treatment show no significant contribution to incidence rates.

**Supplementary Information:**

The online version contains supplementary material available at 10.1186/s13550-021-00805-7.

## Background

Metastatic castration-resistant prostate cancer (mCRPC) is associated with high disease-specific morbidity and mortality [1]. Therapeutic options prolonging overall survival are limited to second-generation antiandrogens (enzalutamide and abiraterone), sipuleucel-T and potentially myelotoxic treatments, including taxane-based chemotherapy and bone-seeking ^223^Ra-dichloride [2–7]. In recent years, radioligand therapy (RLT) directed at the type II transmembrane glycoprotein prostate-specific membrane antigen (PSMA) has been increasingly adopted as a novel treatment for mCRPC. Small-molecule PSMA inhibitors labeled with beta-emitting ^177^Lutetium, most notably the Glu-urea-based radioligand ^177^Lu-PSMA-617 and ^177^Lu-DOTAGA-(I-y)fk(Sub-KuE), briefly termed ^177^Lu-PSMA-I&T have yielded promising anti-tumoral activity with favorable overall tolerability [8, 9].

Hematological decline is a frequent occurrence in patients with progressive mCRPC and considered a risk factor for poor outcome. Based on evidence derived from peptide receptor radionuclide therapy (PRRT) in neuroendocrine neoplasias, the risk of myelosuppression has been taken into account as dose-limiting factor also in RLT. While descriptive assessment of myelotoxic events has been included in a number of prospective and retrospective trials [9–18], their association with potential predisposing factors remains to be elucidated.

Pretreatment factors implicated in the risk of myelosuppression during radionuclide therapy may include preexisting hematologic impairment, previous myelotoxic therapies and bone tumor burden [19]. Irradiation to the bone marrow during RLT can further add to deterioration of hematopoietic function [20]. However, the impact of RLT-specific variables, including administered treatment activity and cumulative activity, has so far not been investigated.

The aim of this study was to examine incidence, severity and reversibility of myelosuppression in patients undergoing RLT with ^177^Lu-PSMA-617 in a sizable and heterogenous cohort. Predisposing factors, including previous therapies, disease burden, as well as administered activity per cycle and treatment course, were then analyzed regarding their contribution to new onset hematologic adverse events.

## Methods

### Patients

A total of 140 patients were treated with ^177^Lu-PSMA-617 in this retrospective single-center series. Production and administration of ^177^Lu-PSMA-617 were performed in accordance with legal regulations set out in the German Drug Registration and Administration Act (AMG § 13 2b). Inclusion criteria for RLT mandated that patients have histologically proven, non-resectable, metastatic prostate cancer with disease progression under standard treatment. Indications were confirmed by an interdisciplinary team including board-certified nuclear medicine physicians, urologists, radiation oncologists, pathologists and oncologists. Sufficient PSMA expression in target lesions was defined as an uptake exceeding the liver uptake on ^68^ Ga-PSMA-11 PET/CT imaging, i.e., scores 2 and 3 according to EANM standardized reporting guidelines v1.0 [21]. An estimated glomerular filtration rate (eGFR [based on the Chronic Kidney Disease Epidemiology Collaboration equation]) of > 30 mL/min/1.73 m^2^, hemoglobin ≥ 8.0 g/dL, white blood cells (WBC) ≥ 2.00 × 10^9^/L and platelets ≥ 75 × 10^9^/L were required for treatment initiation. Extent of bone tumor burden on PET/CT imaging was classified based on the Prostate Cancer Molecular Imaging Standardized Evaluation (PROMISE) initiative as well as previous reports and categorized into 1) uni-/oligo-/multifocal (1–20 lesions) or 2) disseminated/diffuse to obtain sufficient group sizes for subsequent analysis [22, 23].

### Administration

PSMA-617 was obtained from ABX GmbH (Radeberg, Germany), and radiolabeling with ^177^LuCl_3_ was carried out as described in detail before [10, 16]. Quality control was overseen by experienced radiochemists and physicians with respective training in the field. ^177^Lu-PSMA-617 was administered by slow intravenous injection over 30–60 s. Infusion of 1000 mL of saline was initiated 30 min before application at a continuous rate of 300 mL/h. With the intention to limit the uptake to the parotid and submandibular, icepacks were locally applied 30 min before therapy and continued for 1 h [24]. All therapies were performed as in-patient procedures at our nuclear medicine therapy ward. As mandated by radiation protection legislation, patients remained hospitalized for a minimum 48 h; median hospitalization was 3 (range 2–5) days per cycle.

### Toxicity assessment

Repeat blood tests of hematological parameters (hemoglobin, white blood cells and platelets) were undertaken at baseline, prior to each therapy cycle, 2–4 weeks after each cycle and in 6–12-week intervals throughout follow-up. Severity of hematologic adverse events was graded based on Common Terminology Criteria for Adverse Events (CTCAE), version 5.0. Grade ≥ 3 toxicities were termed significant.

### Statistical analysis

Results are presented as median with interquartile range (IQR) and mean ± standard deviation for continuous variables. Categorical variables are reported as frequencies with respective percentages. The paired Student’s t-test was used to compare intraindividual changes in hematologic parameters. Logistic regression analyses were undertaken to explore risk factors relevant for hematological decline. Analysis was carried out per patient (patient-based) and per cycle (cycle-based). Significant hematologic toxicity was defined as an increase in toxicity to grade 3 or higher during the course of RLT and transformed into a dichotomized variable. First, logistic regression analysis was performed for each categorical risk factor. Odds ratios (OR) and 95% confidence intervals (CI) were calculated. Association of hematologic toxicity with continuous baseline variables and administered activity was analyzed using nonparametric rank correlation (Spearman’s correlation coefficient denoted with r_s_). Statistical analyses were performed with SPSS (version 27.0, IBM, Armonk, NY), and GraphPad Prism (version 9.0.1, GraphPad Software, San Diego, CA) was used to plot graphs. All tests were two-sided with *p*-values < 0.05 denominating statistical significance.

## Results

One hundred forty consecutive patients with mCRPC (median age 72 [IQR 67–78] years) met the eligibility criteria for RLT and underwent treatment at our institution. Patient characteristics at baseline are summarized in Table 1. Upon treatment initiation, 109 (78%) patients had low-grade anemia (85 grade 1, 24 grade 2), 13 (9%) leukopenia (10 grade 1, 3 grade 2) and 15 (11%) thrombocytopenia (13 grade 1, 2 grade 2). Two patients with hemoglobin levels slightly below the inclusion threshold (both 7.7 g/dL) were treated after individual consent and lack of therapeutic alternatives. Patients received a total of 497 cycles of ^177^Lu-PSMA-617 with a mean treatment activity of 6.9 $\pm$ 1.3 GBq given in a median of 3 (IQR 2–5) treatment cycles. RLT cycles were administered at intervals of 4–8 weeks, reaching a mean cumulative activity of 24.6 $\pm$ 15.9 GBq. The median follow-up period was 8 (IQR 4–13) months from the start of treatment.

**Table 1:** Baseline characteristics for 140 patients

	All patients (n = 140)
Age	72 (67–78)
PSA (µg/L)	86 (12–258)
Hemoglobin (g/dL)	11.9 (10.4–13.2)
White blood cells (10^9^/L)	6.4 (5.0–7.8)
Platelets (10^9^/L)	238 (188–286)
eGFR (mL/min/1.73 m^2^)	81.8 (68.0–93.7)
Alkaline phosphatase (U/L)	93 (67–200)
LDH (U/L)	240 (205–303)
Gleason score*	
< 8	42 (34)
≥ 8	82 (66)
ECOG performance status	
0	42 (30)
1	81 (58)
2	17 (12)
Sites of metastases	
Bone	125 (89)
Uni-/oligo-/multifocal	48 (34)
Disseminated/diffuse	77 (55)
Lymph nodes	125 (89)
Visceral	33 (24)
Previous mCRPC therapies	
Abiraterone	87 (62)
Enzalutamide	75 (54)
^223^Radium-dichloride	45 (32)
Docetaxel	70 (50)
Cabazitaxel	27 (19)
Other chemotherapies^†^	7 (5)
EBRT (bone metastases)	49 (35)

Data presented as median with interquartile range (IQR) or n (%)

*ECOG* Eastern Cooperative Oncology Group,* PSA* prostate-specific antigen,* eGFR* estimated glomerular filtration rate,* LDH* lactate dehydrogenase,* EBRT* external beam radiotherapy

*For available patients (n = 124), †: cisplatin, 5-FU, carboplatin, mitoxandrone

### Hematologic laboratory values and adverse events

**Fig. 1:**
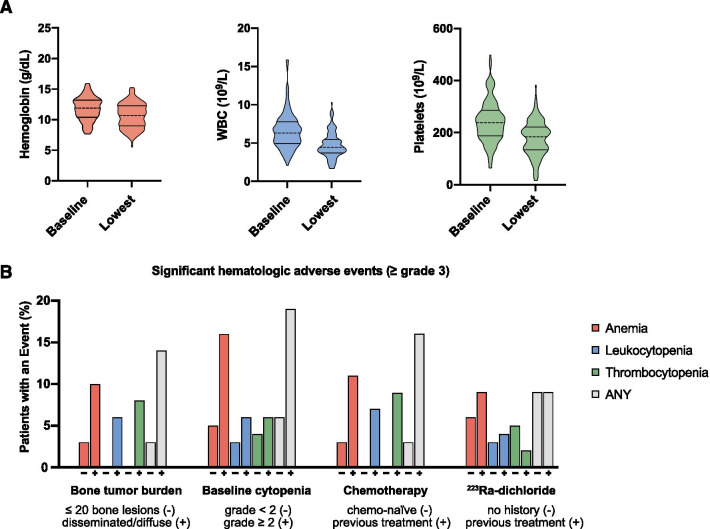
Violin plots for hemoglobin, white blood cell counts (WBC), and platelets at baseline and upon maximum deterioration (**A**). Incidence of grade ≥ 3 hematologic adverse events by risk factor: extent of bone tumor burden with 1) none, uni-/oligo-/multifocal (≤ 20) or 2) disseminated and diffuse bone metastases, chemo-naïve or after previous taxane-based chemotherapy, patients previously receiving ^223^Ra-dichloride or patients with previous hematological decline (CTCAE grade) (**B**)

Hematological parameters showed a slight but significant absolute decline through the course of RLT (Fig. 1A). Median hemoglobin decreased from 11.8 (IQR 10.4–13.2) g/dL at baseline to 10.7 (IQR 9.0–12.3) g/dL at the maximum level of deterioration (*p* < 0.001); median WBC counts shifted from 6.35 (IQR 4.97–7.82) × 10^9^/L to 4.49 (IQR 3.76–5.52) × 10^9^/L (*p* < 0.001) and thrombocytes from 238 (IQR 188–286) × 10^9^/L to 184 (IQR 134–222) × 10^9^/L (p < 0.001).

Significant hematologic adverse events (grade ≥ 3) during RLT occurred in 13 (9.3%) patients, with anemia in 10 (7.1%), leukopenia in 5 (3.6%) and thrombocytopenia in 6 (4.3%), as shown in Table 2. Median cumulative activity prior to grade ≥ 3 toxicity was 20.7 (IQR 7.4–29.6) GBq. Of 13 patients affected by significant hematologic toxicity, 11 (85%) had initially presented with disseminated or diffuse osseous involvement, 6 (46%) with initial grade 2 cytopenia, 11 (85%) had a history of taxane-based chemotherapy, and 4 (31%) had undergone ^223^Ra-dichloride prior to RLT (Fig. 1A). The four patients with more than one cell line affected (2 with bicytopenia and 2 with pancytopenia) all had grade ≥ 1 myelosuppression at treatment initiation. Of 497 cycles administered, 17 (3.4%) were subject to subsequent grade ≥ 3 toxicity, which occurred within a median of 6 weeks after administration. Throughout the follow-up period, no case of late onset severe myelosuppression or myelodysplastic syndrome (MDS) was observed.

**Table 2:** Baseline and intra-/posttherapeutic hematologic toxicity grades based on CTCAE v5.0

	Baseline (%)				Intra-/posttherapeutic (%)			
	Grade 1	Grade 2	Grade 3	Grade 4	Grade 1	Grade 2	Grade 3	Grade 4
Anemia	85 (61)	24 (17)	2 (1)	0 (0)	77 (55)	42 (30)	10 (7)	0 (0)
Leukopenia	10 (7)	3 (2)	0 (0)	0 (0)	27 (19)	11 (8)	5 (4)	0 (0)
Thrombocytopenia	13 (9)	2 (1)	0 (0)	0 (0)	39 (28)	3 (2)	5 (4)	1 (1)

### Course of patients with significant toxicity

Three out of 13 patients with grade ≥ 3 hematologic toxicity spontaneously recovered to lower levels (grade ≤ 2) within 4 to 6 weeks. Nine (69%) patients with significant myelosuppression received transfusion therapy, eight of which were transfused with packed red blood cells and two received platelet concentrates (Table 3). Four (31%) patients could receive additional cycles of RLT either after spontaneous recovery or blood transfusion. Cytopenia was successfully managed in 10 patients. Two patients who experienced significant disease progression following their last cycle died briefly thereafter; one patient was lost to further follow-up. Of the two aforementioned study patients with grade 3 anemia upon treatment initiation, one spontaneously recovered to grade 2 after responding to RLT and one received packed red blood cells throughout the course of RLT and remained at stable grade 2 hemoglobin levels prior to discontinuing RLT due to disease progression after two cycles.

**Table 3:** Previous therapies and course of 13 patients with grade ≥ 3 hematologic adverse events

Patient	Previous therapies	Toxicity (CTC_max_)			Reversibility	Time to toxicity (weeks)	Time to reversibility (weeks)	Course of treatment/disease
		Hb	WBC	Platelets				
1	RP, Rx, ADT, DOCE, ABI, CABA	3	3	3	Yes	8	12	Recovery after transfusion (2xRBC)
2	ADT, DOCE, Ra-223	3	1	0	Yes	4	4	Recovery after transfusion (2xRBC)
3	ADT, local Rx	3	2	0	Yes	4	6	Transfusion therapy, disease progression, death 18 months after PSMA therapy
4	RP, ADT, DOCE, ENZA	2	1	3	–	8	–	Lost to follow-up
5	ADT, bicalutamide, ABI, DOCE, Rx	3	0	0	Yes	6	4	Spontaneous recovery, discontinuation of RLT due to PD, continuation of ABI
6	ADT, ABI, Rx, DOCE, bicalutamide, ENZA	3	3	3	Yes	8	8	Recovery of all cell lines after 4xRBC, diagnosed with NSCLC after PR under RLT
7	RARP, Rx, ADT, ABSI, DOCE, ENZA	3	1	0	Yes	4	6	Continuation of RLT after transfusion (2xRBC)
8	ADT, bicalutamide, Ra-223	3	2	1	Yes	8	4	Carbamazepin intoxication, discontinuation of RLT, spontaneous recovery
9	ADT, palliative Rx, ABI, ENZA, DOCE, CABA, 5-FU	1	1	3	Yes	3	6	Continuation of RLT after spontaneous recovery of platelet count, 3 more cycles, PD
10	RP, ADT, DOCE, ABI, ENZA	3	2	1	Yes	8	8	Continuation of RLT after transfusion (2xRBC)
11	RP, salvage Rx, ADT, DOCE, Ra-223, ABI	3	3	2	Yes	4	12	Transfusion therapy in 4 week intervals (2 × 2 RBC)
12	RARP, Rx, ADT, DOCE, ABI, CABA, carboplatin, etoposid, mitoxandrone, 5-FU	1	3	3	No	8	–	Transfusion of thrombocytes (2xBP), hepatic disease progression
13	RP, salvage Rx, ADT, Ra-223, ABI	3	0	4	No	7	–	Transfusion (RBC, BP) disease progression, death 8 weeks after last cycle

*RP* radical prostatectomy, *RARP* robot-assisted radical prostatectomy, *Rx* radiotherapy, *Ra-223* radium-223-dichloride, *ADT* androgen deprivation therapy, *DOCE* docetaxel, *CABA* cabazitaxel, *ABI* abiraterone, *ENZA* enzalutamide, *NSCLC* non-small cell lung cancer, *RBC* packed red blood cells, *BP* blood platelet concentrates, *Hb* hemoglobin, *WBC* white blood cells, *Plt* platelets

### Analysis of predisposing factors for hematologic adverse events

**Fig. 2:**
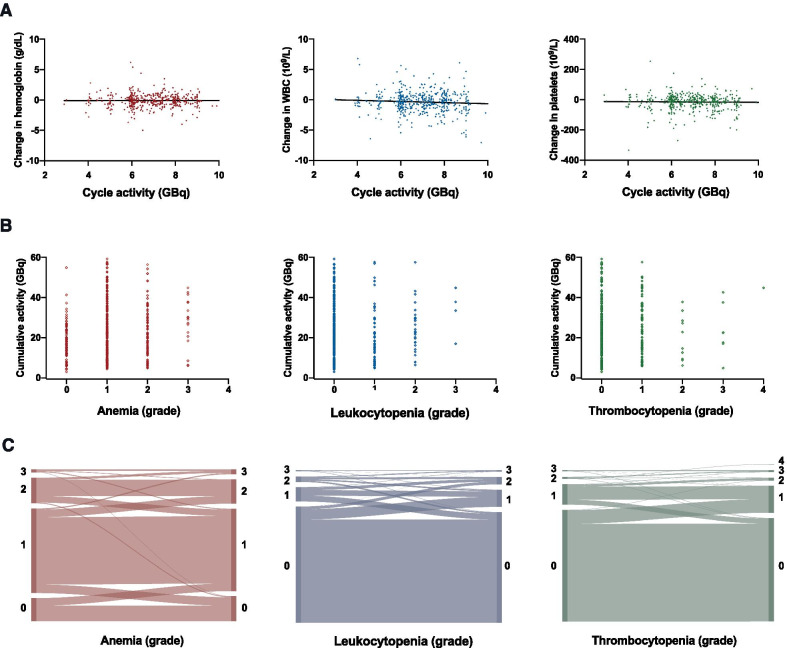
Cycle-based analysis (n = 497): (**A**) association of absolute change in hemoglobin, white blood cells (WBC) and platelets after each treatment cycle with cycle activity, linear trendline, (**B**) association of toxicity grade after each treatment cycle and cumulative treatment activity, (**C**) Sankey diagrams for change in CTC grade after each treatment cycle

Baseline parameters significantly associated with occurrence of grade ≥ 3 toxicities were: high bone tumor burden (no/one/ ≤ 3 vs. disseminated/diffuse bone metastases, odds ratio [OR]: 5.08, 95% confidence interval [95%CI] 1.08–23.86, *p* = 0.04), previous treatment with taxane-based chemotherapy (OR: 4.62, 95%CI 1.23–17.28, *p* = 0.02) and pre-existing grade 2 cytopenia in either cell line (OR: 3.50, 95%CI 1.08–11.32, *p* = 0.04). Previous treatment with ^223^Ra-dichloride (OR: 0.93, 95%CI 0.27–3.20, *p* = 0.58) and presence of bone metastases per se were not significantly associated with occurrence of hematotoxicity (OR: 1.48, 95%CI 0.13–9.22, *p* = 0.71) (Fig. 1B). Of laboratory values assessed at baseline, alkaline phosphatase (ALP) was correlated with grade ≥ 3 toxicities (r_s_ = 0.23, *p* = 0.01) and a moderate inverse correlation of eGFR with the occurrence of grade ≥ 3 thrombopenia was observed (r_s_ = -0.19, *p* = 0.03). Treatment activity per cycle and administered cumulative activity preceding hematotoxic adverse events showed no significant association with incidence of ≥ grade 3 myelosuppression (*p* = 0.29, 0.32) (Fig. 2B, C, Table 4).

**Table 4:** Association of significant hematologic toxicity and potential risk factors

	Significant toxicity (grade ≥ 3)											
	Any			Anemia			Leukopenia			Thrombocytopenia		
	OR	95%CI	p	OR	95%CI	p	OR	95%CI	p	OR	95%CI	p
A												
Bone metastases												
Presence	1.49	0.18–12.32	0.71	1.09	0.13–9.22	0.94	–	–	–	–	–	–
Disseminated/diffuse	5.08	1.08–23.86	0.04	3.54	0.73–17.29	0.12	–	–	–	–	–	–
Baseline hematologic values												
Grade ≥ 2 myelosuppression	3.50	1.08–11.32	0.04	4.00	1.08–14.85	0.04	2.44	0.39–15.28	0.34	1.81	0.32–10.38	0.51
Previous mCRPC therapies												
Taxane-based chemotherapy	4.62	1.23–17.28	0.02	3.45	0.86–13.93	0.08	10.7	0.93–124.74	0.06	11.37	1.11–116.98	0.04
^223^Ra-dichloride	0.93	0.27–3.20	0.91	1.45	0.39–5.41	0.58	1.43	0.23–8.85	0.70	0.43	0.46–3.61	0.42
EBRT (bone metastasis)	2.36	0.75–7.67	0.14	1.96	0.54–7.11	0.31	2.90	0.47–17.99	0.25	1.91	0.37–9.58	0.44

	Significant toxicity (grade ≥ 3)											
	Any		Anemia		Leukopenia		Thrombocytopenia					
	r_s_	*p*	r_s_	*p*	r_s_	*p*	r_s_	*p*				
B												
Baseline laboratory												
ALP	0.23	0.01	0.17	0.05	0.19	0.03	0.19	0.03				
LDH	0.16	0.07	0.10	0.25	0.14	0.11	0.16	0.06				
eGFR	–0.07	0.40	–0.17	0.84	–0.14	0.09	–0.19	0.03				
Administered activity												
Cumulative	0.09	0.32	0.08	0.33	0.05	0.54	0.11	0.21				
Per cycle	–0.05	0.29	–0.07	0.12	0.01	0.85	–0.03	0.58				

(A) Univariate logistic regression. OR: odds ratio, 95%CI: 95% confidence interval and (B) non-parametric rank test, r_s_ = Spearman’s correlation coefficient

## Discussion

In the presented retrospective study, 140 patients receiving RLT for mCRPC were assessed for occurrence of hematologic adverse events and the role of contributing factors. Significant (grade ≥ 3) hematologic toxicity occurred in 3.4% (17/497) of all treatment cycles and in 9.3% (13/140) of all patients undergoing RLT. Hematologic adverse events were manageable and most likely to occur in patients with extensive bone tumor burden (disseminated or diffuse, OR: 5.08, 95%CI 1.08–23.86, *p* = 0.04). Patients with baseline myelosuppression (grade ≥ 2 cytopenia OR: 3.50, 95%CI 1.08–11.32, *p* = 0.04) or previous taxane-based chemotherapy (OR: 4.62, 95%CI 1.23–17.28, *p* = 0.02) also had higher odds of experiencing grade ≥ 3 myelosuppression.

Available taxane-based chemotherapeutic agents for progressive mCRPC bear a risk hematotoxicity, especially to white blood cells. The phase 3 TAX 327 trial for docetaxel yielded grade ≥ 3 neutropenia in 32%; the TROPIC trial reported grades ≥ 3 leukopenia in 68% of patients receiving cabazitaxel [3, 4, 25]. Myelosuppression is also a known side effect in radionuclide therapy [26]. Toxic effects to hematopoietic cells are mediated by both blood-driven recirculating ß-irradiation and scatter radiation from bone metastases. Long-standing experience from peptide receptor radionuclide therapy (PRRT) in neuroendocrine neoplasias (NEN) with ^177^Lu-labeled DOTA^0^-Tyr^3^-octreotate (^177^Lu-DOTATATE) yielded moderate grade ≥ 3 hematotoxicity rates in the range of 8 to 11.3% [26–29]. Beyond the emergence of subacute toxicity, myelodysplastic syndrome (MDS) may develop as a rare, but severe long-term sequel after PRRT in 1–2% of all patients treated [26, 30]. Apart from effects attributable to diverging biokinetics, it may be hypothesized that MDS is less likely observed after ^177^Lu-PSMA-617 due to shorter survival of patients with mCRPC as compared to NEN.

Initial radioimmunological approaches targeting an extracellular PSMA epitope were limited by high rates of myelotoxicity related to the longer plasma half-life inherent to circulating antibodies [31]. In a phase 2 study with the ^177^Lu-labeled monoclonal antibody J591 conducted by Tagawa et al., 47% of all patients developed grade 4 thrombocytopenia necessitating aggressive management and transfusion therapy in 30% of all patients enrolled [32, 33]. Following the advent of the small-molecule ligands ^177^Lu-PSMA-617 and ^177^Lu-PSMA-I&T multiple studies, predominantly within compassionate use programs have included assessment of hematologic adverse events in heterogeneous mCRPC cohorts. Reported overall incidence rates are summarized in Table 5 [9–18, 34–43]. The landmark phase 2 trial conducted by Hofman et al. in 30 patients receiving ^177^Lu-PSMA-617 reported grade ≥ 3 anemia, neutropenia and thrombocytopenia in 13%, 37% and 4% [8]. Results from a large retrospective study by Heck et al. in 100 patients receiving 317 cycles of ^177^Lu-PSMA I&T indicated lower rates, with grade ≥ 3 anemia, neutropenia and thrombocytopenia in 9%, 6% and 6%, respectively [9]. It has to be acknowledged that comparison of white blood cell toxicity in our study was impeded by the fact that differential blood counts for neutrophils and lymphocytes were not available for analysis in all our patients. Recently, Barber et al. contributed a comparative retrospective study using both ^177^Lu-PSMA-617 and ^177^Lu-PSMA I&T in 83 patients previously treated with taxane-based chemotherapy and 84 taxane-naïve controls. Grade ≥ 3 anemia, leukopenia and thrombocytopenia occurred in 8% vs. 1%, 2 vs. 0% and 4% vs. 1% of all study patients [35]. The latter findings indicate an adverse impact of previous taxane-based chemotherapy on subsequent hematotoxcity during RLT, as described in our cohort. In the most recent randomized, multicentric phase 2 trial by Hofman et al. (TheraP, ANZUP 1603) previous treatment with docetaxel was an inclusion criterion. Here, grade ≥ 3 anemia, leukopenia and thrombocytopenia occurred in 8%, 1% and 11% of 98 patients receiving ^177^Lu-PSMA-617, as compared to 8%, 1% and 0% in the standard-of-care arm (n = 85) treated with cabazitaxel [44].

**Table 5:** ^177^Lu-PSMA studies reporting hematologic adverse events

Study	Ref	n	Cycles	Ligand	Activity (GBq/cycle, range)	Anemia grade 1/2	Leukopenia/*neutropenia grade 1/2	Thrombocytopenia grade 1/2	Anemia grade 3/4	Leukopenia/*neutropenia grade 3/4	Thrombocytopenia grade 3/4
Ahmadzadehfar et al. 2015	[10]	10	10	PSMA-617	5.6	1 (10%)	3 (30%)	2 (20%)	1 (10%)	0	0
Ahmadzadehfar et al. 2016	[11]	24	46	PSMA-617	6 (4.1–7.1)	7 (29%)	5 (21%)	4 (17%)	2 (8%)	0	0
Barber et al. 2019	[35]	84/83	n/a	PSMA-617,-I&T	6.3 (5.7–6.8)	81(96%)/73(88%)	22(26%)/23(28%)	21(25%)/27(33%)	1(1%)/7(8%)	0/2(2%)	1(1%)/3(4%)
Baum et al. 2016	[12]	56	125	PSMA-I&T	5.8	n/a	n/a	n/a	0	0	0
Bräuer et al. 2017	[13]	59	159	PSMA-617	6.1 (5.9–6.3)	n/a	n/a	n/a	11 (19%)	2 (4%)	2 (4%)
Derlin et al. 2020	[34]	31/40	n/a	PSMA-617	6.0–7.4	9 (29%)/6 (15%)	5 (16%)/4(10%)	3(10%)/4(10%)	0	0	0
Emmett et al. 2018	[36]	14	n/a	PSMA-617	6.0–8.0	2 (14%)	n/a	1/14 (7%)	0	0	0
Fendler et al. 2016	[14]	30	30	PSMA-617	3.7 or 6	n/a	n/a	n/a	1 (3%)	3 (3%)	0
Heck et al. 2016	[15]	22	43	PSMA-I&T	3.7–7.4	3 (14%)	1*(5%)	5 (23%)	0	0	0
Heck et al. 2018	[9]	100	317	PSMA-I&T	7.4	n/a	n/a	n/a	9 (9%)	6* (6%)	4 (4%)
Hofman et al. 2018	[8]	30	86	PSMA-617	7.5	4 (13%)	11* (37%)	8 (27%)	4 (13%)	11* (37%)	4 (13%)
Hofman et al. 2021	[44]	98	n/a	PSMA-617	6.0–8.5	19 (19%)	10 (10%)	18 (18%)	8 (8%)	1 (1%)	11 (11%)
Kratochwil et al. 2016	[16]	30	70	PSMA-617	3.7–6.0	9 (30%)	8 (27%)	4 (18%)	1 (5%)	0	1 (5%)
Paganelli et al. 2020	[37]	43	n/a	PSMA-617	3.7–5.5	28 (65%)	3*(6.9%)	3(6.9%)	2 (4.8%)	0	0
Rahbar et al. 2016	[18]	74	74	PSMA-617	6.0	26 (35%)	9 (12%)	16 (22%)	1(1%)	0	1 (1%)
Rahbar et al. 2016	[38]	28	50	PSMA-617	5.9	3 (14%)	3 (14%)	5 (23%)	0	0	0
Rathke et al. 2018	[39]	40	120	PSMA-617	4–9.3	0	8 (20%)	2 (5%)	0	1 (3%)	2 (5%)
Scarpa et al. 2017	[40]	10	29	PSMA-617	6 (5.4–6.5)	n/a	n/a	n/a	0	0	0
Seifert et al. 2020	[41]	37/41	n/a	PSMA-617	6.0/7.5	(35%)/(27%)	(49%)/(49%)	(41%)/(34%)	(22%)/(24%)	(8%)/(2%)	(8%)/(2%)
Violet et al. 2019	[42]	50	n/a	PSMA-617	7.5	9 (18%)	12* (24%)	14 (28%)	5 (10%)	3* (6%)	5 (10%)
Yadav et al. 2016	[43]	31	65	PSMA-617	1.1–7.4	9 (31%)	n/a	1 (3%)	1 (3%)	n/a	0
Yadav et al. 2020	[48]	90	281	PSMA-617	1.1–7.8	72 (78%)	10 (11%)	14 (16%)	2 (2%)	1 (1%)	1 (1%)

*n/a* Not available

We report new onset grade ≥ 3 anemia, leukopenia and thrombopenia in 7% (10/140), 4% (5/140) and 4% (6/140) of patients, respectively. Despite delimiting pre-existing cytopenia from therapy-emergent toxicity, differentiation of hematologic decline due to disease progression from true therapy-emergent toxicity remains challenging due to frequently overlapping phenomena. For a conservative estimate, we considered all new onset grade ≥ 3 toxicities in our analysis, regardless of disease progression being a likely contributing factor in a number of cases. Overall, our results appear well in line with data from the foregoing retrospective and prospective studies, taking into account the significant portion of patients with extensive tumor burden and baseline low-grade myelosuppression in the examined cohort.

Our study points toward an influence of predisposing factors on emergence of grade ≥ 3 hematologic adverse events, including taxane-based chemotherapy and initial grade 2 cytopenia. This may be explained by DNA damage conferred by cytotoxic agents [45]. In addition, sequential failure on multiple systemic treatments preceding RLT puts patients at higher odds of developing hematologic decline through disease progression over time. In their post hoc hematologic safety analysis of the ALSYMPCA trial, Vogelzang et al. report both previous taxane-based chemotherapy with docetaxel and baseline cytopenia (anemia and thrombocytopenia) to be associated with grade ≥ 2 thrombocytopenia in mCRPC patients undergoing ^223^Ra-dichloride [19]. Interestingly, the placebo arm also contained relevant rates of new onset toxicity with grade ≥ 3 anemia, neutropenia and thrombocytopenia in 14%, 1% and 3%, underlining the notion that the natural course of mCRPC itself is linked to significant deterioration of bone marrow reserve. In further accordance with our observations in RLT, increased tumor burden (defined as ≥ 6 metastases) was also a predictive factor for hematotoxicity in mCRPC patients receiving ^223^Ra-dichloride.

In our cohort, a slight trend toward grade ≥ 3 hematologic toxicities, especially thrombocytopenia, was observed with decreasing eGFR values. This effect has been described also in PRRT and attributed to decreased plasma clearance of recirculating radionuclides in chronic kidney disease [28, 46].

Cumulative activity and individual treatment activity play a distinct role in defining appropriate regimens for RLT, and various RLT-schemes have been put forth. Rathke et al. clustered 40 patients into treatment groups receiving 4, 6, 7.4 or 9.3 GBq of ^177^Lu-PSMA-617 reporting comparable safety and efficacy, while pointing out a lower mean platelet count in the 10 patients having received 9.3 GBq [39]. Our treatment routine allowed for individual dose adaptation and yielded no correlation between higher treatment activities or high cumulative activities with increased rates of hematologic adverse events. Potential bias must be considered when interpreting the bivariate association of treatment activity and hematotoxic events since myelosuppression was one reason for individual dose de-escalation.

A major limitation to the conducted analysis is undoubtably its retrospective design. The presented patient population is highly heterogenous and may differ from previously reported series, taking into account that both a fraction of patients omitting prior chemotherapy after interdisciplinary counseling and a considerable number of patients with wide-spread bone tumor burden were included in our analysis. Prospective phase 3 data are much anticipated, with results from the VISION trial expected in near future [47].

## Conclusions

Our findings suggest that repeated cycles of RLT with ^177^Lu-PSMA-617 can be carried out at acceptable rates of myelosuppression with cytopenia being most frequently reversible, especially in earlier phases of disease progression. High bone tumor burden, previous taxane-based chemotherapy and initial hematologic decline are possible risk factors for developing significant new onset hematologic adverse events. Administered activity per cycle and cumulative activity had in turn no significant impact. These results call for further refining individualized treatment based on given risk factors for hematologic toxicity.

## Supplementary Information

**Additional file 1: Fig. S1** Maximum intensity projections of ^68^ Ga-PMSA imaging at baseline: (**a**) 81-year-old patient (P8 in Table 3) with limited extent of bone metastases (category 1), the patient developed reversible grade 3 anemia after RLT. (**b**) 75-year-old patient (P 13 in Table 3) with diffuse bone marrow involvement (category 2), developing progressive disease and irreversible hematological decline with grade 3 anemia and grade 4 thrombocytopenia after 6 cycles of RLT.

## Data Availability

The datasets used and/or analyzed during the current study are available from the corresponding author on reasonable request.

## Abbreviations

ALP: Alkaline phosphatase
CI: Confidence interval
CTCAE: Common Terminology Criteria for Adverse Events
EBRT: External beam radiotherapy
ECOG: Eastern Cooperative Oncology Group
eGFR: Estimated glomerular filtration rate
GBq: Gigabecquerel
IQR: Interquartile range
LDH: Lactate dehydrogenase
mCRPC: Metastatic castration-resistant prostate cancer
MDS: Myelodysplastic syndrome
NEN: Neuroendocrine neoplasia
OR: Odds ratio
PROMISE: Prostate Cancer Molecular Imaging Standardized Evaluation
PRRT: Peptide receptor radionuclide therapy
PSA: Prostate-specific antigen
PSMA: Prostate-specific membrane antigen
RLT: Radioligand therapy
WBC: White blood cells
