# Supplementary figures and images for: Hematologic safety of 177Lu-PSMA-617 radioligand therapy in patients with metastatic castration-resistant prostate cancer
Source: EJNMMI Res. 2021 Jul 3;11:61. doi: 10.1186/s13550-021-00805-7 (PMC8254689; doi:10.1186/s13550-021-00805-7)

# FIGURE S1

**A**

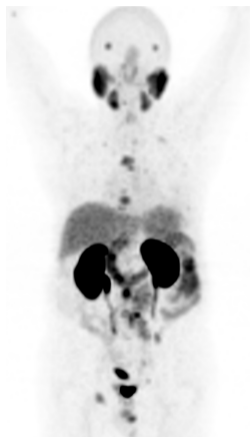

**Category 1**  
( $\leq 20$  bone lesions)

**B**

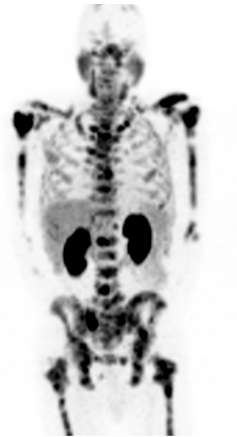

**Category 2**  
(disseminated/diffuse)

Supplement: Supplementary file 1 — Additional file 1: Fig. S1 Maximum intensity projections of 68 Ga-PMSA imaging at baseline: (a) 81-year-old patient (P8 in Table 3) with limited extent of bone metastases (category 1), the patient developed reversible grade 3 anemia after RLT. (b) 75-year-old patient (P 13 in Table 3) with diffuse bone marrow involvement (category 2), developing progressive disease and irreversible hematological decline with grade 3 anemia and grade 4 thrombocytopenia after 6 cycles of RLT. [file 13550_2021_805_MOESM1_ESM.pdf]
